# Knowledge graph-enhanced deep learning for pharmaceutical demand forecasting

**DOI:** 10.1038/s41598-026-35113-4

**Published:** 2026-01-06

**Authors:** Xiaofang Chen, Gang Lu, Hao Zhang, Junmin Wan

**Affiliations:** 1 School of Management, Wuhan University of Technology, Wuhan, 430070 China; 2 Research Institute of Digital Governance and Management Decision Innovation, Wuhan University of Technology, Wuhan, 430070 China; 3 Faculty of Economics, Fukuoka University, Fukuoka, 814-0180 Japan

**Keywords:** Forecast, Gcn, Lstm, Knowledge graph, Pharmaceutical demand, Computational biology and bioinformatics, Health care, Mathematics and computing

## Abstract

Accurate pharmaceutical demand forecasting is essential to ensure timely drug availability, reduce inventory costs, and improve operational efficiency in healthcare supply chains. However, existing statistical, machine learning, and deep learning approaches often struggle to capture the nonlinear and dynamic demand patterns arising from drug substitutions, comorbidity treatments, and seasonal disease fluctuations. To address this challenge, we propose KG-GCN-LSTM, a novel hybrid model that integrates a pharmaceutical knowledge graph (KG) with deep learning techniques. A clipped Graph Convolutional Network (GCN) is employed to extract feature representations from both the historical demand of the target drug and the related drugs encoded in the knowledge graph. The outputs of the GCN are subsequently processed by a Long Short-Term Memory (LSTM) network to capture temporal dynamics in drug demand. Experiments on real-world pharmacy sales data demonstrate that KG-GCN-LSTM consistently outperforms established benchmarks—including ARIMA, SVR, XGBoost, RNN, CNN-LSTM, TimeMixer and NBEATS, achieving a 3.62% reduction in Symmetric Mean Absolute Percentage Error (SMAPE) relative to NBEATS, while delivering performance comparable to the state-of-the-art TimeMixer. These results highlight the effectiveness of knowledge graph–enhanced deep learning in improving the accuracy and robustness of pharmaceutical demand forecasting, which can support data-driven decision-making in healthcare supply chain management.

## Introduction

 In recent years, accurate forecasting of pharmaceutical demand has become increasingly important for the management of healthcare supply chains. With rising medical needs and continuing uncertainties in drug supply, reliable demand prediction helps prevent stockouts, reduce unnecessary inventory, and ensure timely access to essential medicines. It also contributes to improving the responsiveness and operational performance of the supply chain. However, forecasting drug demand remains a difficult task because pharmaceutical consumption patterns are often affected by many interacting factors, including seasonal disease cycles, the emergence of new illnesses, policy or regulatory adjustments, and substitution behaviors among drugs^1,2^.

Traditional time-series models, such as Autoregressive Integrated Moving Average (ARIMA) and exponential smoothing, usually face limitations when dealing with these complex patterns. These models assume relatively stable or linear trends and often treat each drug as an independent time series. As a result, they fail to consider important relationships such as co-prescription, therapeutic equivalence, and substitution, all of which can significantly influence demand fluctuations. In recent years, machine learning and deep learning methods have shown stronger capabilities in capturing nonlinear and dynamic patterns. Models based on recurrent neural network (LSTM and GRU) and convolutional neural network (CNN) have demonstrated improved performance by learning long-term temporal dependencies and extracting local patterns from time-series data. LSTM^3^, in particular, is well suited for modeling long-term variations, while CNN can effectively identify short-term fluctuations and periodic behaviors.

Despite these advantages, purely sequential models still have difficulty capturing the relational information among different drugs. Drug substitution and combination usage naturally form a graph structure, and ignoring this structure may lead to incomplete modeling of the underlying demand interactions. Graph convolutional network (GCN^4^provide a promising way to address this issue by learning representations from graph-structured data. Several recent studies have reported improvements in predictive accuracy when incorporating GCNs into forecasting tasks. Shi, et al^5^. proposes an integrated GCN-LSTM model for predicting stock price movement by assuming that large fluctuations are primarily caused by high-volume trades among interrelated stocks. By modeling capital flows through multi-relation graphs and combining GCN and LSTM, the approach significantly improves prediction accuracy. Yang, et al^6^. proposes a GCN-LSTM model to accurately predict urban rooftop PV potential by incorporating spatial shading between buildings, addressing the limitations of traditional geospatial and time-series methods. Wei, et al^7^. proposes a spatiotemporal power load forecasting method that combines improved scale-limited dynamic time warping (LDTW) clustering with a GCN-LSTM model to better capture temporal and spatial correlations in grid data.

Motivated by these findings, this paper proposes a new forecasting framework, KG-GCN-LSTM, which integrates a pharmaceutical knowledge graph with a GCN-LSTM^8^ model to jointly capture relational and temporal dependencies. We construct a pharmaceutical knowledge graph to represent substitution and combination relationships between drugs. A clipped GCN is applied to learn relational features based on the graph structure, and these features are then fed into an LSTM network for multi-step demand forecasting. This hybrid design allows the model to leverage both inter-drug relationships and time-series dynamics within a unified architecture, thereby improving the accuracy and robustness of pharmaceutical demand forecasting in practical supply chain scenarios.

Developing the KG-GCN-LSTM model involves several challenges:

1. Pharmaceutical demand is influenced by many complex and nonlinear factors, resulting in high variability across time.
2. Different related drugs may exert different levels of influence on the target drug’s demand, making it difficult to determine appropriate relational weights.
3. It is necessary to integrate relational information and nonlinear temporal patterns at the same time, which increases the technical complexity of model design.

To address these challenges and develop an effective forecasting framework, the main contributions of this work are summarized as follows:

1. We construct a pharmaceutical knowledge graph to represent the relationships among drugs and symptoms. Ablation results show that relational information contributes substantially to the performance improvement of the model.
2. We develop a hybrid GCN–LSTM architecture that jointly models inter-drug dependencies and temporal dynamics, and apply a clipping operation to the GCN so that it concentrates more effectively on learning features related to the prediction target, thereby enabling more accurate demand forecasts.
3. Using a real-world pharmaceutical sales dataset, we conduct extensive experiments demonstrating that the proposed method outperforms traditional statistical models and state-of-the-art deep learning approaches across various evaluation metrics.

The remainder of this paper is organized as follows: Sect. “Literature review” reviews related work on demand forecasting. Section “Methods” presents the proposed KG-GCN-LSTM framework in detail. Section “Graph data construction via knowledge graph” describes the experimental setup and discusses the results. Finally, Sect. “GCN-Based relational embedding” concludes the paper and outlines directions for future research.

## Literature review

Demand forecasting has long been recognized as a crucial component of supply chain management. Traditional statistical models such as ARIMA, Exponential Smoothing, and Seasonal Trend Decomposition have been widely adopted due to their interpretability and simplicity. Gilbert^9^ applied the ARIMA model to predict consumer demand, while Kareem and Majeed^10^used the Seasonal ARIMA (SARIMA) model to forecast monthly peak electricity load. Jian, et al^11^. utilized an ARIMA model to accurately forecast the number of chronic kidney disease (CKD) patients and estimate the associated economic burden in China.

However, these approaches, including ARIMA and exponential smoothing, often fall short when dealing with the high variability and non-linear patterns commonly observed in demand data, particularly when demand is influenced by external, seasonal, and behavioral factors.

Driven by Industry 4.0 and advancements in artificial intelligence, machine learning (ML) techniques such as Random Forest, eXtreme Gradient Boosting (XGBoost), and Support Vector Regression (SVR) have gained widespread attention. Vairagade, et al^12^. employed a Random Forest model to predict demand for goods in the supply chain. Ji, et al^13^. applied the XGBoost model to forecast future sales demand for an e-commerce company. Yani and Aamer^14^ empirically demonstrated that Random Forest and decision-tree-based models outperformed traditional baselines by 10–41% in pharmaceutical demand forecasting. Machine learning models offer several advantages, including the ability to capture complex non-linear relationships, incorporate a wide range of features, and adapt to large, high-dimensional datasets. However, they also present challenges such as interpretability issues, overfitting risks, and sensitivity to data preprocessing^15^. Yildiz and Sucuoglu^16^develops a low-cost, real-time IoT-based air quality forecasting system enhanced with machine learning, achieving high predictive accuracy and contributing to sustainable urban environmental management. Cao, et al^17^. proposes a hybrid daily peak load forecasting model combining XGBoost and Multiple Linear Regression (MLR), achieving significantly higher prediction accuracy with substantial reductions in RMSE and MAPE.

In recent years, deep learning models have gained significant attention for time series forecasting, particularly in demand prediction. Recurrent Neural Network (RNN), especially Long Short-Term Memory (LSTM) networks, have shown superior performance over traditional models in capturing temporal dependencies^18^. DeepAR, an autoregressive recurrent model, further advances this field by modeling the conditional distribution of future values and has achieved notable success in large-scale retail forecasting.

Several studies have explored hybrid architectures to enhance forecasting accuracy. For instance, Punia, et al^19^. combined LSTM with Random Forest to capture both temporal and regression-based relationships in multi-channel retail data. Oreshkin, et al^20^. propose N-BEATS, a deep residual fully connected architecture for univariate time-series forecasting, demonstrating the effectiveness of generic deep learning models in forecasting tasks. Zhang, et al^21^. showed that an improved SVR/LSTM model with a modified Gorilla Troops optimizer can predict electric load more accurately than existing methods. Jeon and Seong^22^improved DeepAR with distribution-based inputs and model ensembling, ranking third in the M5 competition. Zha, et al^23^. applied CNN-LSTM for gas field production forecasting. Tian, et al^24^. showed that a spatiotemporal analysis and Bi-GRU model can more effectively predict urban electrical carbon emissions. Farhadi, et al^25^. proposes a hybrid LSTM–GRU model that integrates comprehensive technical analysis features and weighted feature learning to jointly capture short- and long-term dependencies in stock price dynamics. Recently, a notable advancement is TimeMixer^26^, which proposes a fully MLP-based architecture combined with a past-decomposable-mixing mechanism. By decoupling multi-scale temporal variations, TimeMixer achieves competitive long-term forecasting performance while maintaining significantly lower computational complexity.

In the pharmaceutical sector, recent studies have shown that machine learning and deep learning techniques can significantly improve the accuracy of drug demand forecasting compared with traditional time-series models^27,28^. Rathipriya, et al^29^. found shallow neural networks more effective than deep models across diverse drug categories. Hapsari^30^utilized K-Means clustering with Bi-LSTM to forecast pharmaceutical demand, outperforming traditional machine learning approaches. Similarly, Mbonyinshuti, et al^31^. and Priyadharshini, et al^32^. demonstrated the advantages of LSTM-based and CNN-LSTM hybrid models in improving medicine demand forecasting and inventory optimization in healthcare systems.

Deep learning models such as CNN, LSTM and TimeMixer have demonstrated significant advantages in demand forecasting, particularly in effectively capturing diverse temporal and spatial patterns within complex time series data. However, these models often overlook the interaction information embedded in graph structures—for instance, the compositional or substitutive relationships among pharmaceuticals, or the interdependencies between financial assets. To enhance the representational capacity of forecasting models, recent studies have begun integrating domain-specific knowledge through graph-based structures^7,33,34^. In healthcare, GCN have been used for disease prediction, drug repurposing, medicine recommendation^35^ and patient risk stratification^36^, Li, et al^37^. develops a dynamic knowledge graph–based elderly care system that integrates personal, medical, and nursing data to support report generation, risk identification, and intelligent services, achieving favorable expert evaluation results.Yang, et al^38^. conducts a bibliometric analysis of global “AI + chem” research from 2000 to 2024, highlighting the emerging role of knowledge graphs in organizing chemical data and revealing research trends in molecular design, reaction prediction, and materials innovation. Although numerous studies have demonstrated the applications of medical knowledge graphs, to the best of the authors’ knowledge, no existing research has leveraged a pharmaceutical knowledge graph to enhance drug demand forecasting.

Drug demand is influenced by a variety of factors, such as weather conditions, regional characteristics, population structure, and price fluctuations. Equally important, however, are the substitution and combination relationships that exist among pharmaceuticals. For example, the antibiotic amoxicillin can be substituted with cefalexin, and in the treatment of the common cold, clinicians often prescribe antibiotics together with vitamin C to promote faster recovery. These inter-drug relationships can substantially influence demand patterns across the pharmaceutical market. To better leverage the multi-relational information contained in pharmaceutical knowledge graphs and further enhance forecasting performance, this study proposes a knowledge graph–enhanced drug demand forecasting model (KG-GCN-LSTM). The model integrates inter-drug relational dependencies with temporal demand dynamics, thereby improving the accuracy of demand predictions. As shown in Table 1, while previous methods address one or two of these aspects, our proposed model is the only one that effectively integrates all four dimensions, particularly the incorporation of knowledge graphs to enhance pharmaceutical demand forecasting.

**Table 1:** Comparison of related works and the proposed model across different dimensions.

Method	Non-linear modeling	Temporal dependency	Spatial correlation	Knowledge graph integration
ARIMA^11^	×	√	×	×
SVR^21^	√	√	×	×
XGBoost^17^	√	√	×	×
DeepAR^22^	√	√	×	×
LSTM^19^/Bi-LSTM^30^	√	√	×	×
CNN-LSTM^32^	√	√	√	×
NBEATS^20^	√	√	×	×
TimeMixer^26^	√	√	×	×
Ours (KG-GCN-LSTM)	√	√	√	√

## Methods

In this section, we propose a hybrid prediction framework that effectively integrates structural semantic information from a drug knowledge graph with temporal demand patterns by combining a GCN and a LSTM network. A drug knowledge graph (KG) is a structured representation of pharmaceutical domain knowledge, where entities such as drugs, symptoms, and diseases are modeled as nodes, and their semantic or functional relationships (substitution or co-prescription) are represented as edges. This graph-based representation enables the encoding of domain-specific prior knowledge, which is often overlooked by purely data-driven time-series models. To incorporate this structured knowledge into demand forecasting, we employ GCN to learn low-dimensional embeddings of drugs from the knowledge graph. The GCN propagates and aggregates information across connected nodes, allowing each drug’s representation to reflect not only its intrinsic characteristics but also its relationships with other drugs. These graph-based embeddings are then integrated into the temporal forecasting module (LSTM), enabling the model to jointly capture structural knowledge dependencies and temporal demand dynamics. By combining the knowledge graph with GCN, our KG-GCN-LSTM model effectively leverages both domain knowledge and time-series patterns, resulting in improved prediction accuracy and robustness.

**Fig. 1:**
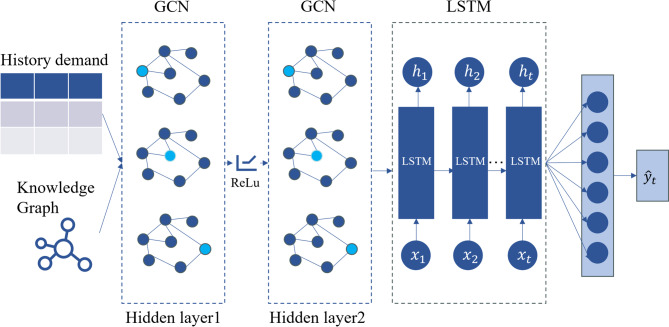
KG-GCN-LSTM model architecture diagram.

As illustrated in Fig. 1, the proposed model architecture is composed of four key components:

### Graph data construction via knowledge graph

A domain-specific drug knowledge graph is constructed by extracting structured semantic relationships among drugs, such as substitution and combination relations. This graph is represented as a heterogeneous network where nodes correspond to drugs and edges represent semantic relations. Historical demand data for each drug are aligned with corresponding graph nodes to form attribute-enhanced graph data for subsequent learning.

### GCN-Based relational embedding

To capture topological dependencies and semantic correlations between drugs, a multi-layer Graph Convolutional Network is employed. The GCN propagates information across the graph structure, generating relational embeddings that encode not only a drug’s own features but also contextual information aggregated from its neighboring nodes (e.g., substitute or complementary drugs). This enables the model to learn high-level inter-drug representations that reflect real-world co-usage or substitution patterns.

### LSTM-based temporal modeling

The temporal dynamics of drug demand are modeled using an LSTM network, which is well-suited for learning sequential dependencies and long-term temporal patterns. The historical demand sequences of each drug, enriched with relational embeddings, are fed into the LSTM to learn time-aware representations that capture seasonality, trends, and irregular demand fluctuations.

## Prediction

The LSTM module’s outputs are fed via a fully linked layer to obtain the final demand prediction. This fusion layer combines structural knowledge from the drug graph with temporal features from the LSTM, allowing the model to produce more context-aware and accurate forecasts. The output represents a forecast of future demand.

This hybrid architecture allows for a more comprehensive modeling of pharmaceutical demand by unifying relational semantics and temporal dynamics, addressing the limitations of traditional univariate forecasting models that fail to account for complex inter-drug interactions.

### Input data construction

**Fig. 2:**
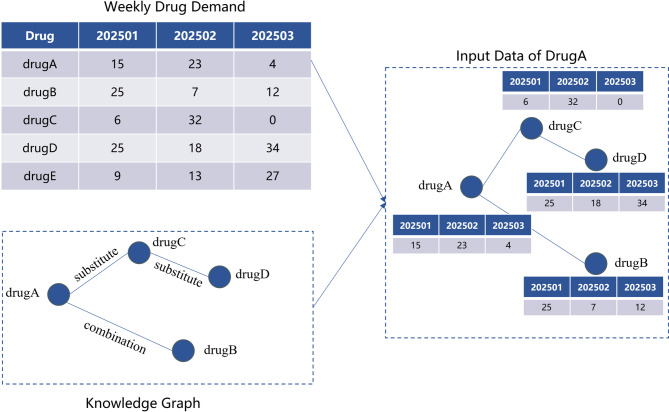
Graph data construction.

As illustrated in Fig. 2, for a given target drug A, the model input is constructed by combining the historical demand data of all drugs with a drug knowledge graph. Specifically, the knowledge graph is queried to identify all drugs that are directly or indirectly related to drug A. For example, drugs B and C may share a direct relationship with drug A, while drug D may be connected through an intermediate node. The historical demand series for these related drugs are then extracted to form the raw input data for the demand forecasting model of drug A.

Subsequently, the raw input data undergoes three preprocessing steps: data cleaning, outlier handling and data assembly.

Data cleaning: Accurate demand forecasting relies heavily on the availability of sufficient and continuous historical demand data. Therefore, during the data preparation stage, products with insufficient sales history were excluded to ensure data reliability and stability. Specifically, pharmaceutical products with fewer than 40 weeks of recorded sales were filtered out, where each week containing at least one sales transaction was counted as one valid sales week. This filtering process helps eliminate sparsely sold or discontinued items that could introduce noise and bias into the training process.

Regarding missing values, all unrecorded sales entries were filled with zero to preserve the temporal consistency of the dataset. This approach reflects the realistic scenario that the absence of sales in a given week typically indicates zero demand rather than missing information. By doing so, the continuity and integrity of the time series are maintained, allowing the model to more accurately capture both active and inactive demand periods. This preprocessing step ensures that the cleaned dataset provides a robust foundation for subsequent feature extraction and model training.

Outlier Handling: To ensure that the model learns the dominant patterns of pharmaceutical demand time series, outlier detection and correction were applied. Specifically, within each time window, any value that deviated from the mean by more than three standard deviations was identified as an outlier. Such anomalous values were then replaced with the corresponding window mean to mitigate their impact on model training. This approach effectively reduces noise and prevents extreme fluctuations from biasing the learning of general demand trends.

Data Assembly: Each sequence of 16 weeks dataset is treated as a single training sample. The window is then shifted forward by one week to generate the next sample. This sliding-window approach continues until the end of the available time series is reached.

Based on the preprocessed data, we construct a drug graph $\:G=(\mathcal{V},\mathcal{E})$, where each node $\:v\in\:\mathcal{V}$ corresponds to a specific drug, and each edge $\:e\in\:\mathcal{E}$ denotes a semantic relationship between drugs. These relationships include, but are not limited to substitution and combination.

### Graph convolutional network

We apply a two-layer clipped GCN to embed the structural information from the drug graph into dense representations. In this two-layer GCN network, the primary focus is on capturing the information of the target drug node itself. Since associated drugs exert varying degrees of influence on the target drug, a weight matrix is employed to learn the respective weights of different associated drugs. Given a drug graph $\:G=(V,E)$ with $\:N$ nodes, let $\:M\in\:{\mathbb{R}}^{N\times\:N}$ denote the adjacency matrix, where $\:{M}_{ij}=1$ if there is an edge between node $\:i$ and node $\:j$, and 0 otherwise; $\:X\in\:{\mathbb{R}}^{N\times\:F}$ is the feature matrix with $\:F$-dimensional input features for each node. To ensure that each node retains its own information during convolution, we introduce self-loops:

1 $$\:\stackrel{\prime }{M}=M+I$$

where $\:I$ is the identity matrix. The corresponding degree matrix is:

2 $$\:{\stackrel{\prime }{D}}_{ii}=\sum\:_{j}\:{\stackrel{\prime }{M}}_{ij}$$

The propagation rule for a single GCN layer is defined as:

3 $$\:{H}^{(l+1)}=\sigma\:\left({\stackrel{\prime }{D}}^{-1/2}\stackrel{\prime }{M}{\stackrel{\prime }{D}}^{-1/2}{H}^{\left(l\right)}{W}^{\left(l\right)}\right)$$

where:

$\:{H}^{\left(l\right)}\in\:{\mathbb{R}}^{N\times\:{d}_{l}}$ is the hidden representation at the $\:l$-th layer, with $\:{H}^{\left(0\right)}=X$.

$\:{W}^{\left(l\right)}\in\:{\mathbb{R}}^{{d}_{l}\times\:{d}_{l+1}}$ is the trainable weight matrix. The weight matrix parameters enable the model to learn the influence weights of related drugs on the target drug being predicted.

$\:\sigma\:(\cdot\:)$ is a nonlinear activation function ReLU.

$\:{\stackrel{\prime }{D}}^{-1/2}\stackrel{\prime }{M}{\stackrel{\prime }{D}}^{-1/2}$ is the symmetrically normalized adjacency matrix, which ensures numerical stability and avoids scaling issues during aggregation.

Through this mechanism, each node updates its feature representation by aggregating information from its neighbors, weighted by graph structure.

**Fig. 3:**
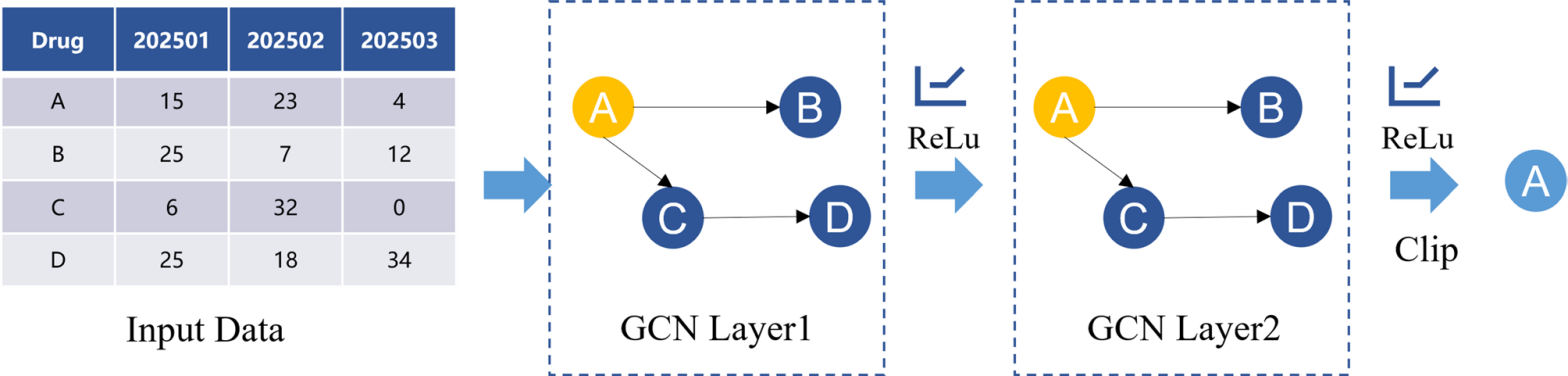
A two-layer GCN architecture.

As illustrated in Fig. 3, a two-layer GCN is adopted to strike a balance between model expressiveness and computational complexity, and it can be formally expressed as:

4 $$\:Z=\sigma\:({\stackrel{\prime }{D}}^{-1/2}\stackrel{\prime }{M}{\stackrel{\prime }{D}}^{-1/2},\sigma\:({\stackrel{\prime }{D}}^{-1/2}\stackrel{\prime }{M}{\stackrel{\prime }{D}}^{-1/2}X{W}^{\left(0\right)}\left){W}^{\left(1\right)}\right)$$

Specifically, in Fig. 3, the input to the GCN consists of the features of drug A and its adjacent nodes B, C, and D. The purpose of the GCN module is to extract the feature information of drug A and its related drugs. The first layer maps the input features into a hidden space, extracting local structural embeddings. The second layer aggregates higher-order dependencies and generates the final embeddings ($\:{Z}_{t}\in\:{\mathbb{R}}^{N*d}$), where (*N* = 4 consist of A, B, C, D). After two layers of GCN processing, the feature representation of drug A already incorporates information from related drugs. Since the prediction target is the demand for drug A, a clip operation is applied to reduce the influence of features from other associated drugs. Specifically, the output layer retains only the feature representation of drug A, while all features corresponding to other drugs are removed at this stage. The GCN output of drug A are subsequently used as input for the LSTM module, which captures temporal dynamics in pharmaceutical demand.

### LSTM

The GCN output vector $\:{Z}_{t}\in\:{\mathbb{R}}^{d}$ is the input of LSTM:

5 $$\:{lstm\_x}_{t}={Z}_{t}$$

**Fig. 4:**
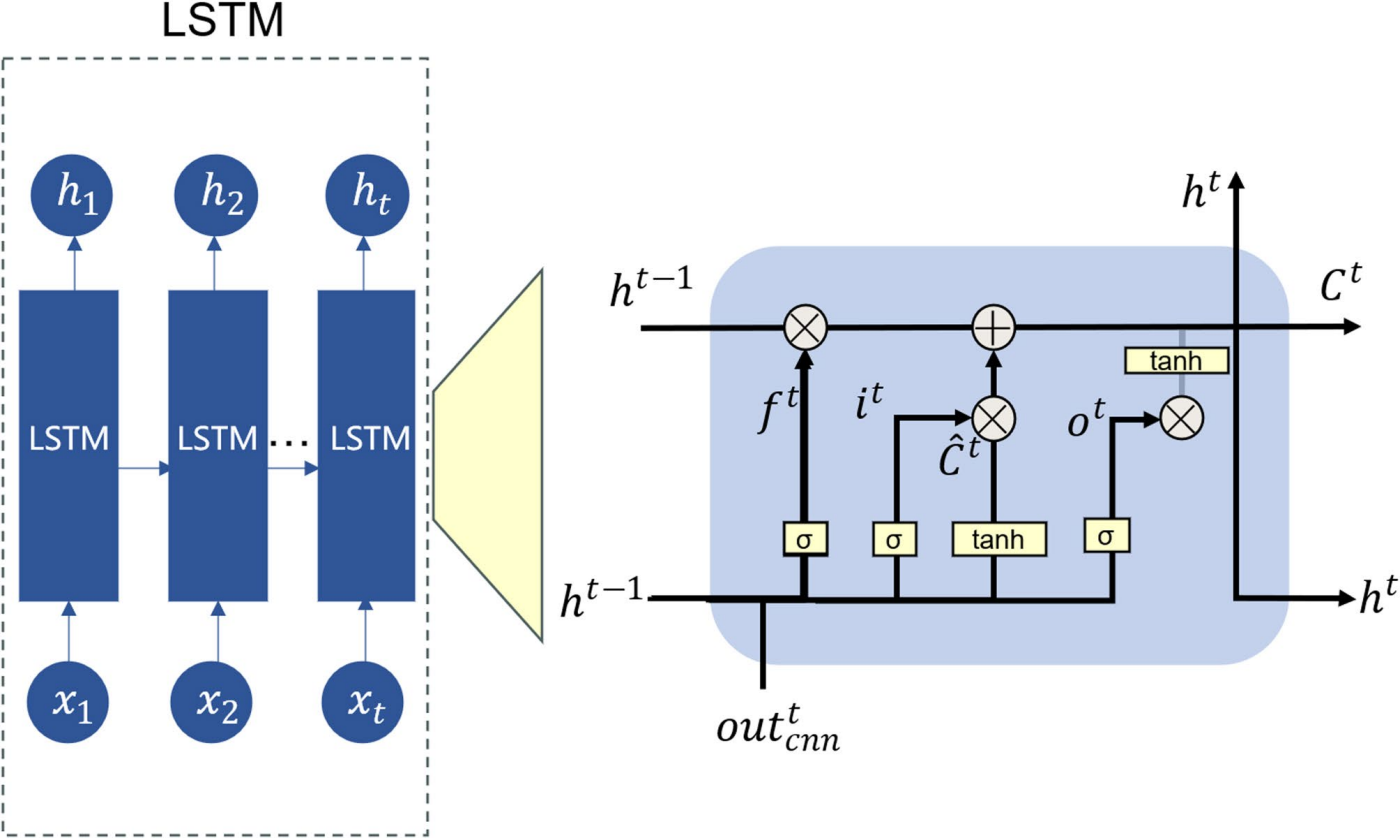
LSTM framework.

The core structure of the LSTM network comprises four key components: the forget gate, input gate, candidate state, and output gate, as illustrated in Fig. 4.

6 $$\:{f}_{t}=\sigma\:\left({W}_{f}\mathrm{*}{x}_{t}+{b}_{f}\right)$$

7 $$\:{i}_{t}=\sigma\:\left({W}_{i}\mathrm{*}{x}_{t}+{b}_{i}\right)$$

8 $$\:{\widehat{C}}^{t}=tanh\left({W}_{c}{\mathrm{*}x}_{t}+{b}_{c}\right)$$

9 $$\:{c}_{t}={f}_{t}\odot\:{c}_{t-1}+{i}_{t}\odot\:{\widehat{C}}^{t}$$

10 $$\:{o}_{t}=\sigma\:\left({W}_{o}{\mathrm{*}x}_{t}+{b}_{o}\right)$$

11 $$\:{h}_{t}={o}_{t}\odot\:tanh\left({c}_{t}\right)$$

The forget gate determines whether the memory from the previous time step should be retained. Its computation is defined by Eq. (6), where $\:{f}_{t}$​ denotes the forget gate output, and $\:\sigma\:(\cdot\:)$ represents the sigmoid activation function, which maps values to the range (0, 1). $\:{W}_{f}$ is the weight matrix, and $\:{b}_{f}$​ is the bias term for the forget gate. The input gate, defined in Eq. (7), controls the extent to which the current input affects the memory cell. Here, $\:{W}_{i}$​ and $\:{b}_{i}$ denote the weight matrix and bias for the input gate, respectively. The candidate cell state is computed as shown in Eq. (8), where $\:{W}_{c}$​ and $\:{b}_{c}$​ represent the corresponding weights and biases, and $\:\odot\:$ denotes element-wise multiplication. The memory cell state is then updated by combining the outputs of the forget gate and input gate, as defined in Eq. (9). Next, the output gate determines which parts of the current cell state are exposed as output. The computation of the output gate is shown in Eq. (10), where $\:{W}_{o}$​ and $\:{b}_{o}$​ refer to the weights and biases of the output gate, respectively. The hidden state $\:{h}_{t}$​ at the current time step is computed as shown in Eq. (11).

### Output

The final demand forecast $\:{\stackrel{\prime }{y}}_{t+\tau\:}$ ​ for a future time step $\:t+\tau\:$ is produced by feeding the last hidden state hth_tht​ of the LSTM into a fully connected Multi-Layer Perceptron (MLP), as expressed in Eq. (12):

12 $$\:{\stackrel{\prime }{y}}_{t+\tau\:}=\mathrm{MLP}\left({h}_{t}\right)$$

This dense layer serves as a regression head that maps the learned temporal features encoded in the hidden state to a scalar prediction representing the forecasted drug demand. The MLP consists of one linear layers with non-linear activation functions ReLU, enabling the model to capture complex non-linear relationships between past hidden states and future demand values.

To train the entire model, including the LSTM and MLP components, we adopt the Mean Squared Error (MSE) as the loss function. This loss is computed across all drugs and all time steps in the training set, as shown in Eq. (13):

13 $$\:\mathcal{L}=\frac{1}{NT}\sum\:_{i=1}^{N}\:\sum\:_{t=1}^{T}\:{\left({y}_{t}^{i}-{\stackrel{\prime }{y}}_{t}^{i}\right)}^{2}$$

Here, $\:N$ denotes the total number of drugs, and $\:T$ is the total number of time steps. $\:{y}_{t}^{i}$​ and $\:{\stackrel{\prime }{y}}_{t}^{i}$​ represent the ground-truth and predicted demand for drug $\:i$ at time $\:t$, respectively. By minimizing this loss function during training via backpropagation, the model learns to reduce the prediction error over time, thereby improving its generalization capability on unseen future demand scenarios.

This end-to-end training framework ensures that both the sequential patterns captured by the LSTM and the non-linear mapping capability of the MLP contribute effectively to accurate drug demand forecasting.

### Pseudocode and parameter

**Fig. 5:**
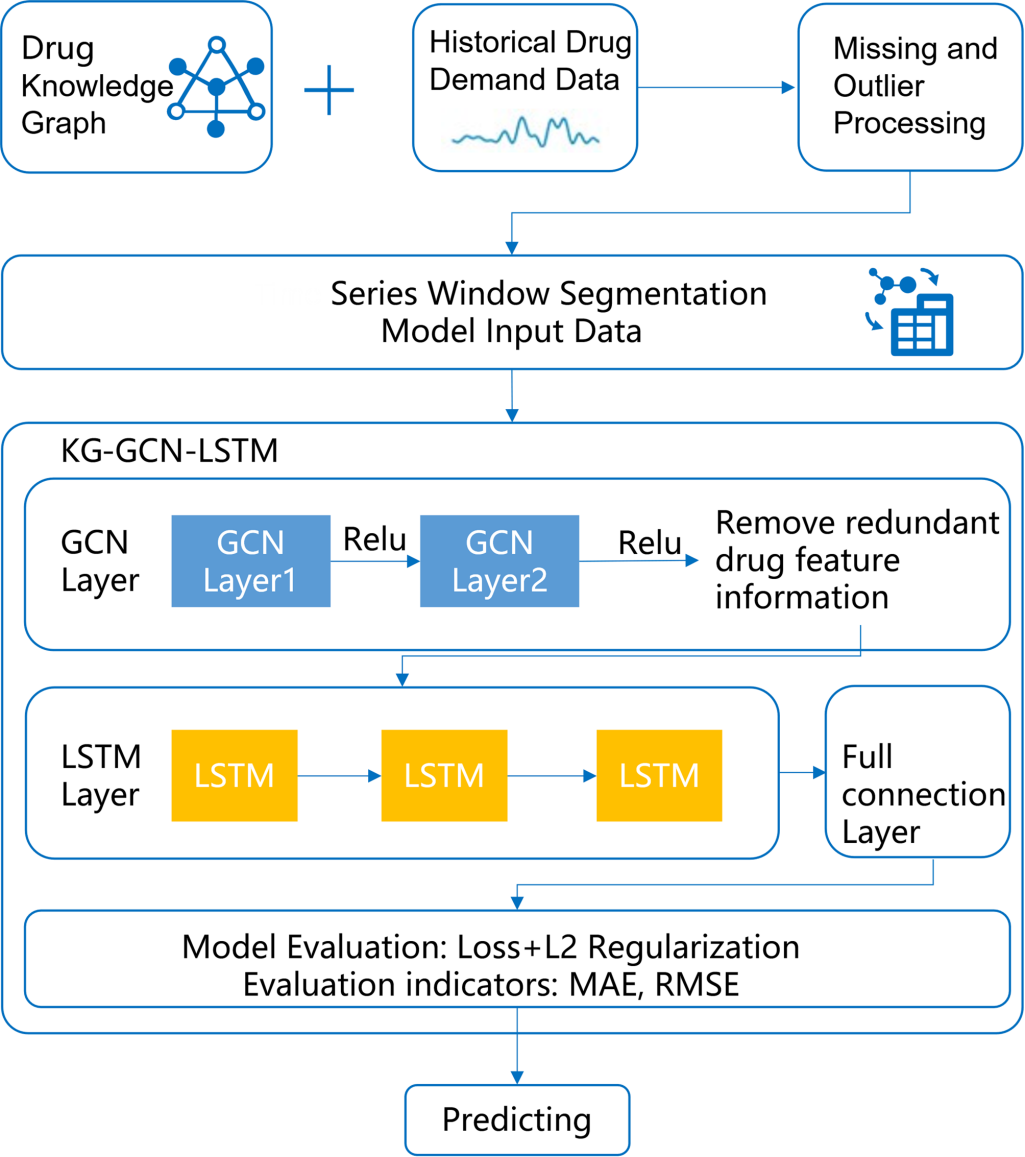
Flowchart of the KG-GCN-LSTM algorithm.

The complete workflow of the KG-GCN-LSTM algorithm is illustrated in the Fig. 5.

**Figure:**
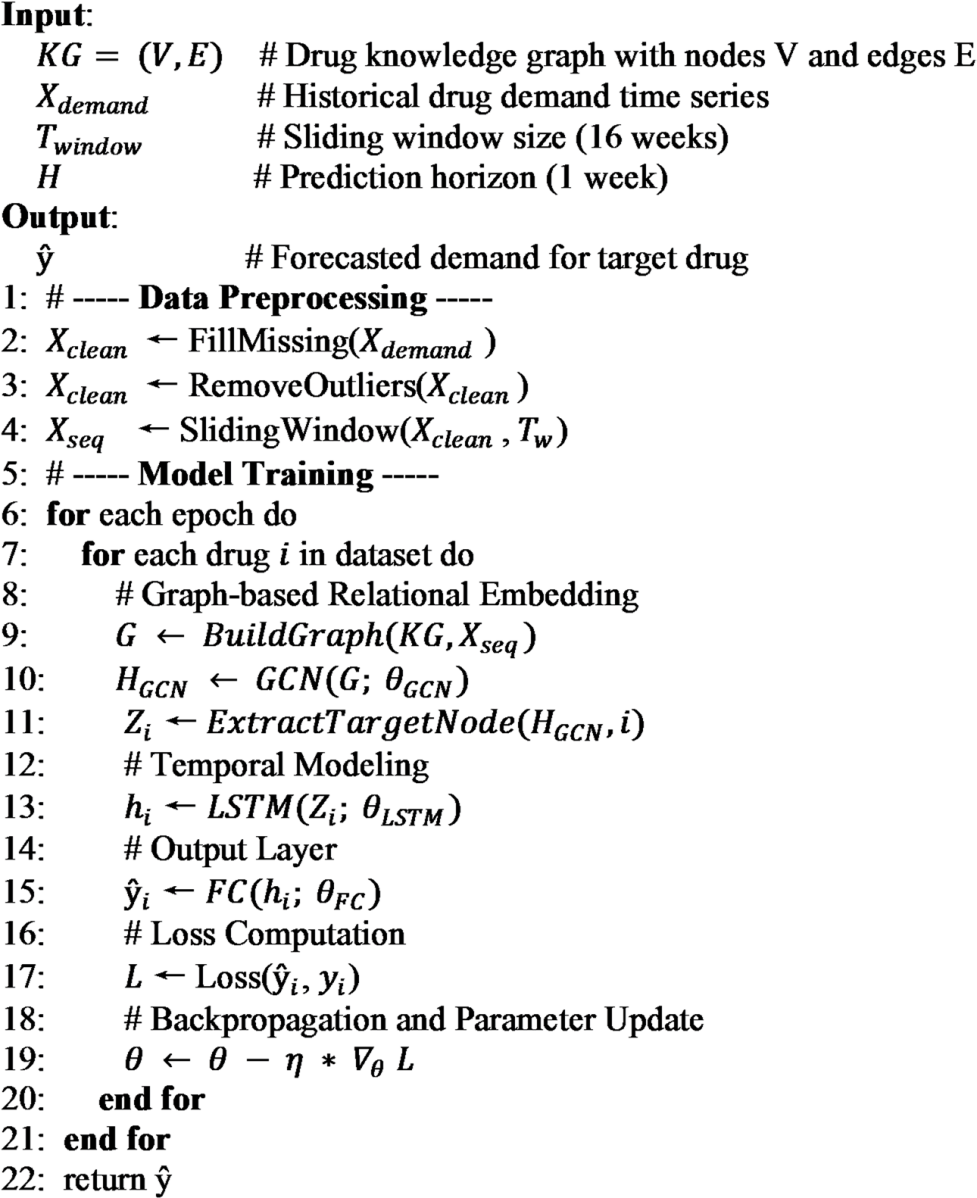
The pseudocode of the algorithm:

The detailed network parameters of the KG-GCN-LSTM model are shown in Table 2, where N denotes the number of input drugs, including the target drug to be predicted and the drugs associated with it in the knowledge graph. The proposed model is implemented using the PyTorch deep learning framework. Model training is conducted using the Adam optimizer. To ensure the optimal performance of KG-GCN-LSTM, we determined the hyperparameters through a grid search strategy based on the performance (SMAPE) on the validation set. The search ranges for key parameters were set as follows:

- Learning Rate: {0.0001, 0.001, 0.005}
- Hidden Dimension: {16, 32, 64}
- Dropout Rate: {0.1, 0.2, 0.3, 0.5}

Based on the experimental results, the optimal configuration was determined to be: a learning rate of 0.001, a hidden dimension of 64, a dropout rate of 0.2. To prevent overfitting and enhance generalization performance, an early stopping strategy is applied, whereby training is halted when the validation loss ceases to improve over 500 epochs. We have conducted experiments on CPU i7 and GPU NVIDIA RTX 3090, achieving an average training time of approximately 6.5 s per epoch, ensuring its feasibility for real-world deployment.

**Table 2:** Network parameter settings of KG-GCN-LSTM.

Module	Layer	Input shape	Output shape	Parameter count
Data input	Historical demand data	$\:(N\:\times\:\:{T}_{w}$)	$\:(N\:\times\:\:{T}_{w}$)	
GCN Block	GCN Layer 1	$\:(N\:\times\:\:{T}_{w}$)	$\:(N\:\times\:\:{T}_{w}\:\times\:\:64)$	1088
GCN Block	GCN Layer 2	$\:(N\:\times\:\:{T}_{w}\:\times\:\:64)$	$\:(N\:\times\:\:{T}_{w}\:\times\:\:64)$	4160
GCN Block	Target Node Extraction	$\:(N\:\times\:\:{T}_{w}\:\times\:\:64)$	($\:{T}_{w}$ × 64)	
LSTM Block	LSTM (1 layer)	($\:{T}_{w}$ × 64)	(1 × 64)	33,024
Fully Connection Layer	Dense Layer	(1 × 64)	(1 × 1)	64

## Results and discussion

The experiments are conducted on a public dataset, the Retail Sales Dataset of a Pharmacy in Indonesia^39^, which contains daily sales records for a variety of pharmaceutical products. The dataset comprises 521,566 pharmaceutical sales records. Due to substantial daily fluctuations in drug sales, the data were aggregated by drug and by week. To ensure data completeness, only drugs with sales records spanning more than 40 weeks were retained, resulting in a final dataset containing weekly sales data for 219 drugs. Among these, 16% (35 items) are classified as medical devices or consumables (e.g., masks) which are treated as isolated nodes due to the lack of chemical interaction properties. The remaining 84% (184 items) are pharmaceutical drugs, which were mapped to DrugBank database^40^ IDs. To maximize the matching accuracy, we performed data cleaning on the source ms_product table in the dataset. Specifically, we extracted the NAMA field (Product Name) and removed the dosage specification and strength information (e.g., removing “500 mg”) from the strings, retaining only the core drug names. Subsequently, we standardized these core names to International Nonproprietary Names (INN) and finally matched them against the DrugBank database.

To construct a knowledge graph that precisely reflects the dependencies within our specific drugs, we determined the final sets of substitution and combination drugs through a strict intersection between the DrugBank databases and our cleaned sales records. Specifically:

- Substitution Set: We leveraged the Anatomical Therapeutic Chemical (ATC) classification system. For each drug, we extracted the Level 4, first 5 characters of ATC code representing the Chemical Subgroup. Drugs sharing the same Level 4 of ATC were considered as substitution drugs.
- Combination Set: We identified combination by retrieving Drug-Drug Interaction (DDI) records from DrugBank.

Finally, the 184 pharmaceutical items were matched to identify 317 substitution relationships and 76 combination relationships. When constructing the knowledge graph, consistent weights were assigned to both substitution and combination edges, enabling the model to automatically learn the corresponding weights.

### Baselines for comparison

To assess the effectiveness of the proposed KG-GCN-LSTM model, we compare KG- GCN-LSTM to the following baselines: (1) auto-ARIMA^41^, (2) SVR^42^, (3) XGBoost^43^, (4) RNN^44^, (5) CNN-LSTM^23^, (6) NBEATS^20^, (7) TimeMixer^26^. Table 3 provides an overview of each model’s characteristics along with their key parameter settings. All models, including baselines and the proposed KG-GCN-LSTM, are trained using the training set, with hyperparameters optimized on the validation set. Specifically, the dataset for each drug was partitioned as follows:

Test Set: The final 8 weeks of the series were reserved for evaluating the model’s performance.

Validation Set: The 4 weeks immediately preceding the test set were used for hyperparameter tuning and prevent overfitting.

Training Set: All remaining historical data prior to the validation period were used for model training.

**Table 3:** Baseline models and key parameters.

Model	Description	Key parameters
auto-ARIMA	ARIMA is a classical time series forecasting model that captures linear dependencies through autoregressive, differencing, and moving average components.	The parameters p, d, and q are selected by minimizing the AIC, with up to 100 iterations.
SVR	SVR is a machine learning regression method based on support vector machines, suitable for small-scale and nonlinear data.	Kernel = RBF, C = 100, gamma = 0.01, epsilon = 0.1
XGBoost	XGBoost is a powerful gradient boosting framework using decision trees, known for its accuracy and handling of complex non-linear patterns.	max_depth = 6, eta = 0.1, subsample = 0.8, n_estimators = 100
RNN	The RNN baseline models sequential dependencies using recurrent hidden states, making it suitable for time series forecasting.	hidden_size = 64, num_layers = 1, dropout = 0.2
CNN-LSTM	CNN-LSTM integrates CNN for short-term feature extraction and LSTM for long-term sequence modeling.	CNN kernel size = 3, filters = 64, LSTM: hidden_size = 64, dropout = 0.2
NBEATS	N-BEATS(Neural Basis Expansion Analysis for Interpretable Time Series) is a deep learning architecture for time series forecasting that employs backward and forward residual blocks to capture both trend and seasonal components in a fully interpretable manner.	number of stacks = 3, block layers = 2, hidden_size = 128, dropout = 0.2, batch size = 256
TimeMixer	TimeMixer is a fully MLP-based time-series forecasting model that captures complex temporal dynamics by disentangling and mixing multiscale seasonal and trend information for accurate and efficient forecasting.	Layers per MLP block: 2 MLP dimension: 256 dropout: 0.1
KG-GCN-LSTM (Proposed Model)	KG-GCN-LSTM combines graph convolutional networks to model relational dependencies between drugs and LSTM to capture temporal dynamics.	GCN: layers = 2, hidden_size = 64, LSTM: hidden_size = 64, dropout = 0.2

Forecasting performance is evaluated using commonly adopted metrics, including Mean Absolute Error (MAE), Root Mean Square Error (RMSE), and Symmetric Mean Absolute Percentage Error (SMAPE), allowing a comprehensive assessment of both absolute and relative prediction accuracy.

MAE measures the average magnitude of the absolute differences between the predicted and actual values, offering a straightforward interpretation of the model’s prediction bias. It is defined as:

14 $$\:MAE=\frac{1}{n}\sum\:_{i=1}^{n}\:\left|{y}_{i}-{\stackrel{\prime }{y}}_{i}\right|$$

where $\:n$ denotes the number of samples, $\:{y}_{i}$​ represents the actual value of the ith sample, and $\:{\stackrel{\prime }{y}}_{i}$​ is the predicted value.

RMSE evaluates the square root of the average squared prediction errors, emphasizing larger errors due to the squaring operation. It is more sensitive to outliers compared to MAE and is given by:

15 $$\:RMSE=\sqrt{\frac{1}{n}\sum\:_{i=1}^{n}\:\:{\left({y}_{i}-{\stackrel{\prime }{y}}_{i}\right)}^{2}}$$

SMAPE quantifies prediction accuracy as a percentage, measuring the average absolute error relative to the actual values, Unlike the traditional Mean Absolute Percentage Error (MAPE), SMAPE addresses the issue of asymmetry by normalizing the absolute error with the average of the actual and predicted values. This makes it particularly useful when the scale of the data varies or when actual values are close to zero:

16 $$\:SMAPE=\frac{100\mathrm{\%}}{n}\sum\:_{i=1}^{n}\:\left|\frac{{y}_{i}-{\stackrel{\prime }{y}}_{i}}{(\left|{y}_{i}\right|+|{\stackrel{\prime }{y}}_{i}\left|\right)/2\:}\right|$$

Together, these metrics offer complementary perspectives on the model’s predictive accuracy, robustness, and practical utility.

**Fig. 6:**
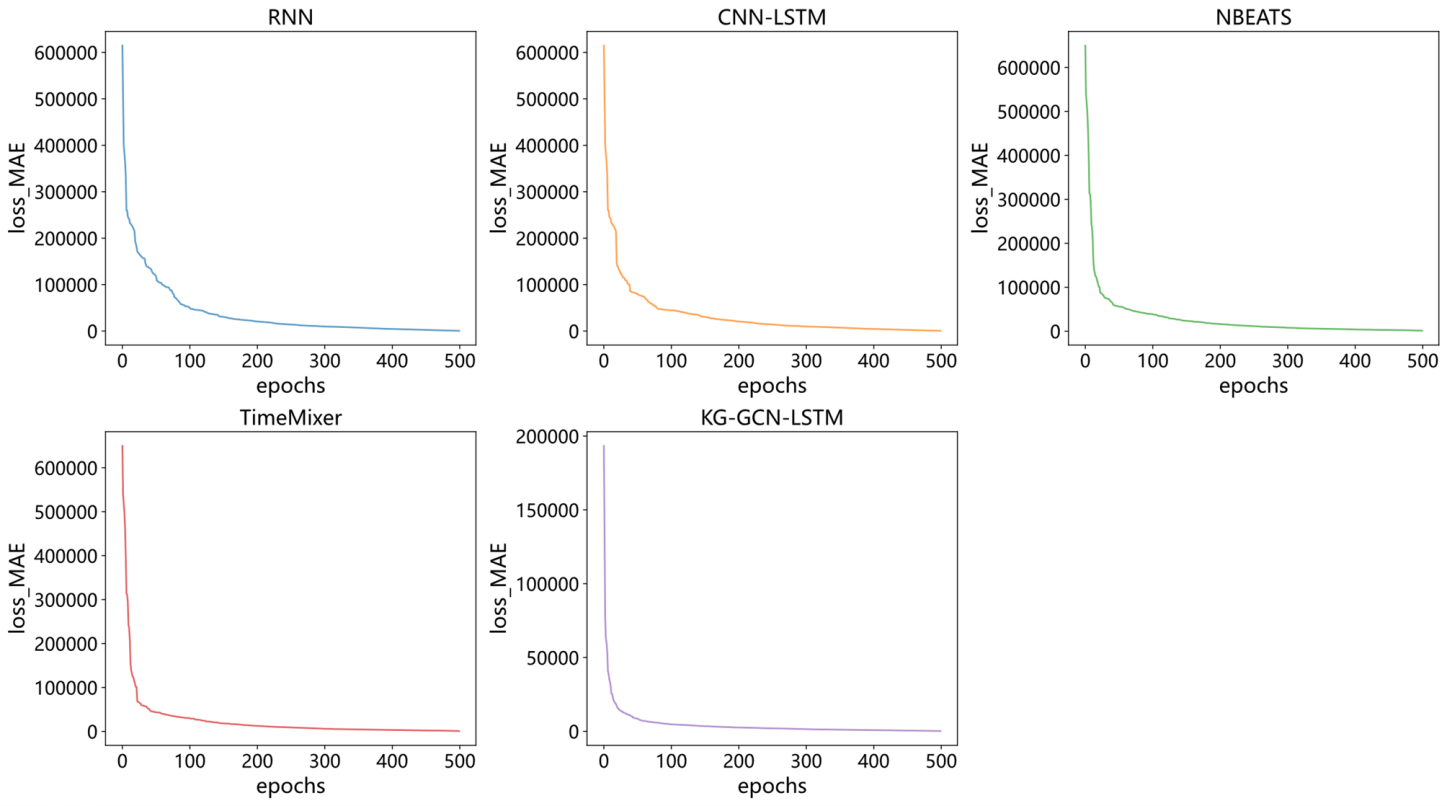
Training loss trajectories of RNN, CNN-LSTM, NBEATS, TimeMixer, and KG-GCN-LSTM over epochs.

To further examine the learning dynamics of different models, the evolution of training errors was analyzed. Since AutoARIMA relies on parameter search rather than iterative optimization, its training error is not analyzed here. For the remaining models, MAE was used as the loss metric. Figure 6 illustrates the training error trajectories of RNN, CNN-LSTM, NBEATS, TimeMixer, and KG-GCN-LSTM. Among these, KG-GCN-LSTM demonstrates the fastest error reduction, converges rapidly to a stable level, and exhibits a smoother loss curve, indicating stronger robustness and training stability. In contrast, the other five models show relatively similar error patterns throughout the training process.

Table 4 presents the comparative forecasting performance of different baseline models and the proposed KG-GCN-LSTM model, evaluated using three standard metrics: MAE, RMSE, and SMAPE. The results indicate significant performance differences across classical statistical models, machine learning methods, and advanced deep learning architectures.

Among the state-of-the-art models, NBEATS surpasses CNN-LSTM in performance but still struggles with relatively high error rates (MAE = 78.2468, RMSE = 96.3988, SMAPE = 11.86%). On the other hand, the proposed KG-GCN-LSTM model outperforms all competitors across the three evaluation metrics, achieving the lowest error values (MAE = 53.9080, RMSE = 65.4058, SMAPE = 8.24%). Remarkably, compared to NBEATS, our model reduces SMAPE by 3.62%, underscoring its superior ability to integrate pharmaceutical knowledge graph features with temporal forecasting. Furthermore, our model achieves performance comparable to the state-of-the-art TimeMixer; specifically, it improves SMAPE by 0.31% compared to TimeMixer, although with a slightly higher RMSE (+ 8.4). Nevertheless, KG-GCN-LSTM offers superior interpretability and is particularly well-suited for cold-start scenarios where historical demand data is unavailable.

These findings validate the significance of incorporating structured pharmaceutical knowledge through graph representations in improving forecasting accuracy. The results also demonstrate that combining domain-specific knowledge with deep sequential models enhances both prediction robustness and adaptability to the inherent complexities of pharmaceutical demand patterns.

**Table 4:** Performance comparison with baseline models, the results are reported as mean standard deviation over 5 independent runs.

Model	MAE	RMSE	SMAPE (%)
AutoARIMA	67.8769 ± 0.00	79.0307 ± 0.00	10.63%
SVR	71.1110 ± 0.00	81.1767 ± 0.00	11.02%
XGBoost	68.1974 ± 0.05	77.6257 ± 0.08	10.56%
RNN	67.3514 ± 1.24	80.8032 ± 1.58	10.68%
CNN-LSTM	82.7919 ± 1.85	96.0930 ± 2.10	12.60%
NBEATS	78.2468 ± 0.92	96.3988 ± 1.15	11.86%
TimeMixer	54.1543 ± 0.58	57.0879 ± 0.65	8.55%
KG-GCN-LSTM	53.9080 ± 0.43	65.4058 ± 0.69	8.24%

**Fig. 7:**
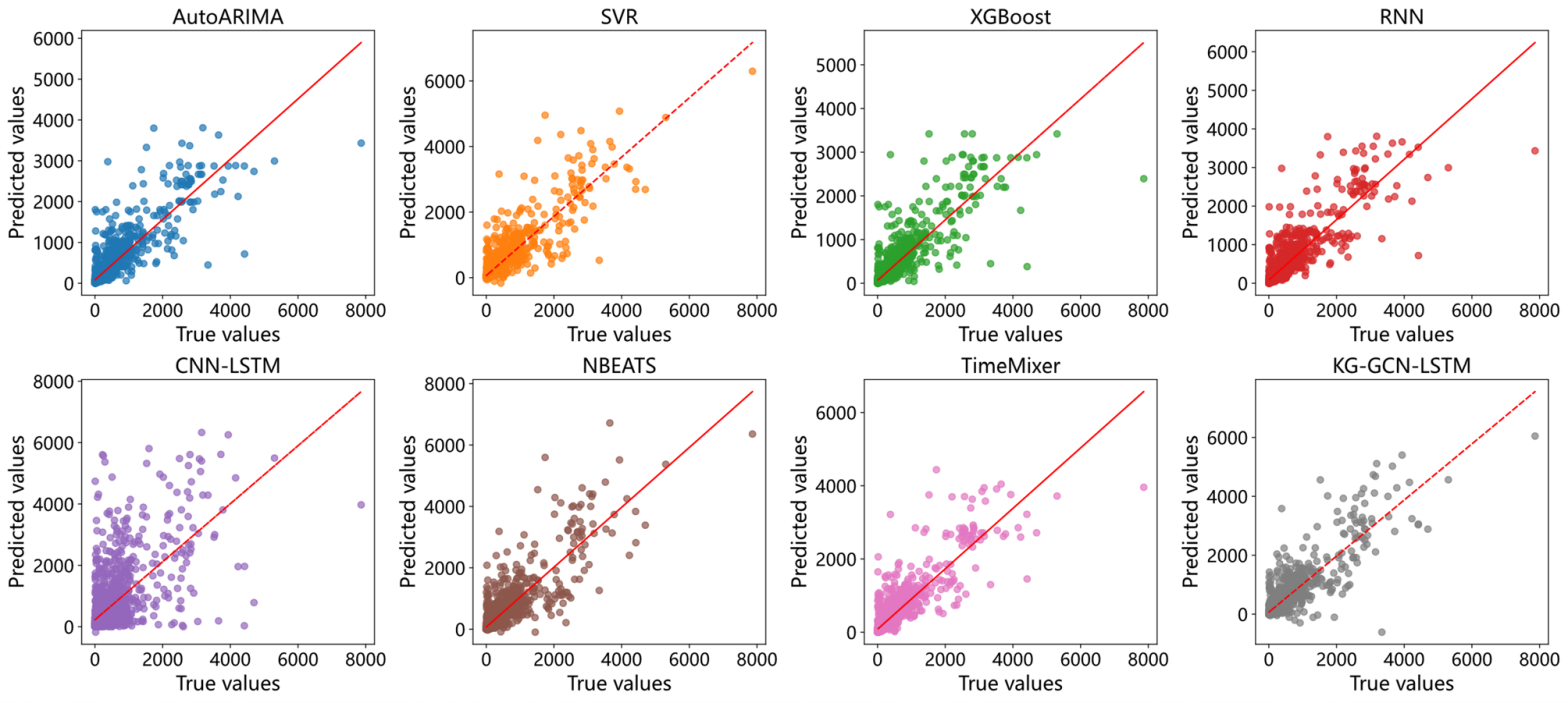
Scatter plots of the predicted and observed during the seven model prediction phases.

Figure 7 presents a comparison between the predicted and actual values. The red line indicates the reference line where the predicted values equal the actual values. The closer the points are to this line, the more accurate the predictions. As shown in Fig. 7, the points corresponding to the KG-GCN-LSTM model are closer to the red line and more densely clustered, indicating higher prediction accuracy. In contrast, the points corresponding to the CNN-LSTM model are more dispersed and lie farther from the reference line, reflecting lower accuracy.

**Fig. 8:**
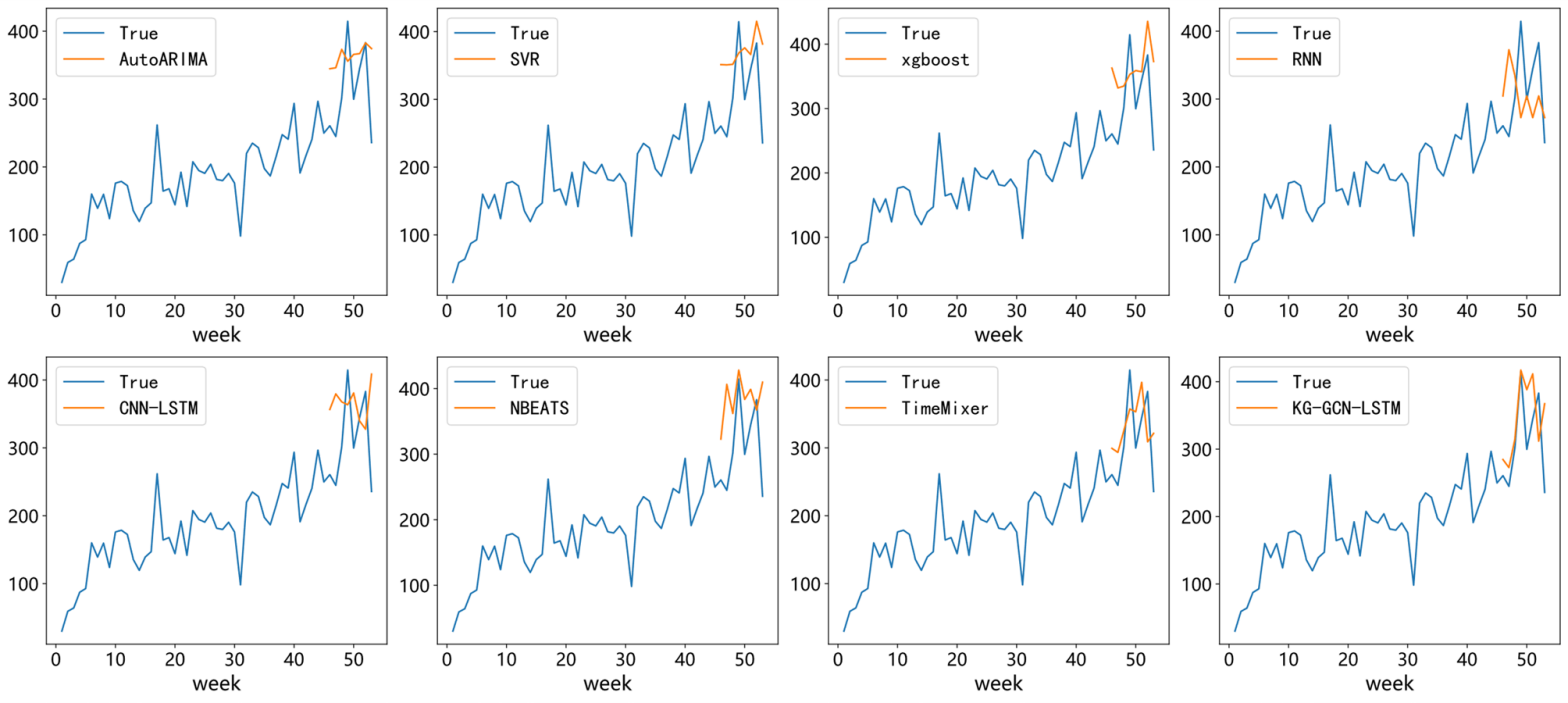
Comparison of true and predicted demand of all modes.

To better understand the predictive capability of our model, Fig. 8 presents a visual comparison of average forecasting results across all models. The time-series plots show that AutoARIMA and RNN capture the general demand trends but often lag behind rapid fluctuations. CNN-LSTM demonstrates unstable predictions with noticeable deviations during peak demand periods, while NBEATS yields smoother curves but still fails to accurately follow local variations in the data.

In contrast, the proposed KG-GCN-LSTM exhibits the closest alignment with the actual demand curve, particularly in capturing sudden demand surges and seasonal fluctuations. This observation is consistent with the quantitative metrics reported in Table 4, where KG-GCN-LSTM achieves the lowest MAE and SMAPE.

The combination of numerical evidence and visual analysis strongly supports that integrating drug knowledge graph representations with temporal modeling allows KG-GCN-LSTM to achieve more accurate and reliable pharmaceutical demand forecasting than both classical methods and state-of-the-art deep learning models.

### Ablation study of different GCN layers and clip

To evaluate the effect of the number of GCN layers and the clipping operation on forecasting performance, we compare models containing 1, 2, and 3 GCN layers (with clipping), as well as a version without clipping. The main purpose of this experiment is to examine whether increasing the GCN depth can help the model capture relational information among drugs and improve the prediction accuracy of the KG-GCN-LSTM model. In addition, to verify the effectiveness of the clipping design, we compare the performance between the two-layer clipped GCN and the two-layer non-clipped GCN.

As shown in Table 4, the model with one GCN layer performs the worst (MAE = 68.3034, RMSE = 77.5396, SMAPE = 10.43%). This result indicates that shallow aggregation cannot sufficiently extract association features between drugs, making it difficult for the model to identify which relational information is more important. The two-layer clipped GCN achieves the best performance (MAE = 53.9080, RMSE = 65.4058, SMAPE = 8.24%). A possible reason is that two layers allow the model to integrate neighborhood information more effectively and capture deeper association patterns, thus producing more discriminative feature representations. The two-layer non-clipped GCN performs worse than all clipped models, which suggests that keeping all relational features introduces interference to demand prediction and that the clipping operation is beneficial for improving model performance. The three-layer GCN performs between the one- and two-layer models, showing that deeper propagation may cause over-encoding of relational information and reduce the model’s ability to represent drug relationships.

**Fig. 9:**
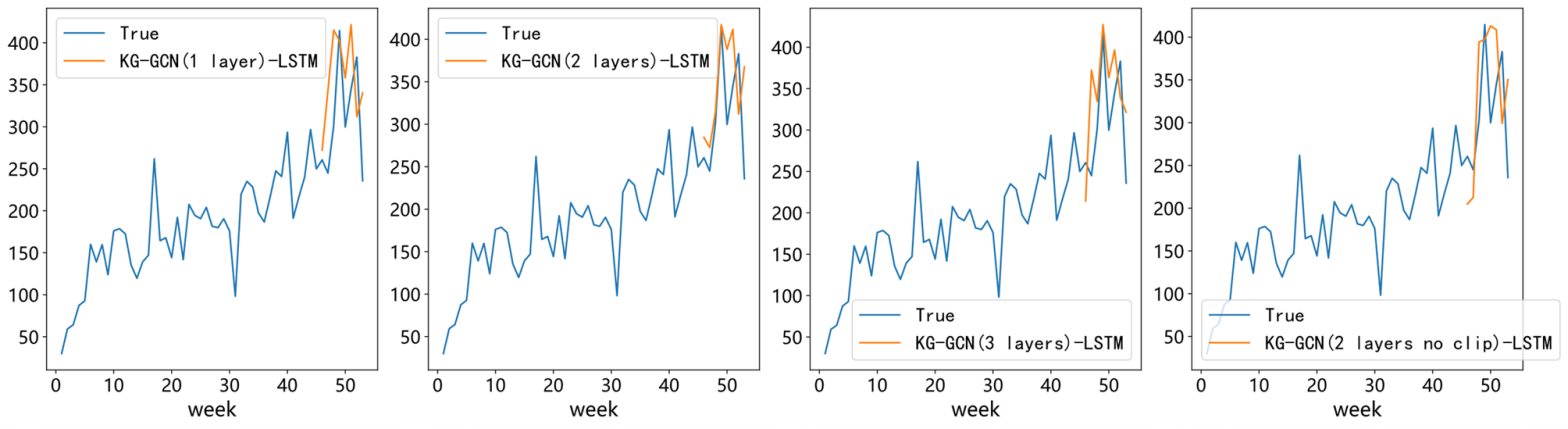
Comparison of true and predicted demand with different GCN layers.

Figure 9 further shows that the overall prediction trends of the four settings are similar, but the two-layer clipped model fits the actual demand curve most closely, which is consistent with the results in Table 5.

In general, the number of GCN layers has a clear influence on forecasting performance. Too few layers lead to insufficient feature learning, while too many layers may cause excessive encoding. A two-layer GCN achieves a good balance by extracting both the target drug’s features and the features of related drugs without over-encoding. Moreover, the clipping operation helps the GCN focus on learning the features related to the prediction target, resulting in more accurate drug demand forecasts.

**Table 5:** Performance comparison of KG-GCN-LSTM with different GCN pool strategy.

Model	MAE	RMSE	SMAPE (%)
GCN(1 layer)	68.3034	77.5396	10.43%
GCN(2 layers)	53.9080	65.4058	8.24%
GCN(2 layers no clip)	71.7713	79.2150	11.34%
GCN(3 layers)	58.0198	66.7277	9.39%

### Ablation study of different LSTM hidden size

To study how the hidden size of the LSTM submodule influences the forecasting results, we tested three different hidden dimensions: 16, 32, and 64. The aim is to check whether changing the hidden size can help the KG-GCN-LSTM model learn more temporal information and nonlinear features, so that the prediction accuracy can be improved.

As shown in Table 6, the model has the worst performance when the hidden size is 16. When we increase the hidden size, the performance becomes better. With a hidden size of 32, the SMAPE is lower than that of size 16 by 0.45%. When the hidden size reaches 64, the SMAPE is further reduced by 1.67% compared with size 32. A larger hidden size allows the model to capture deeper nonlinear relations in the time series, which leads to more accurate prediction.

**Fig. 10:**
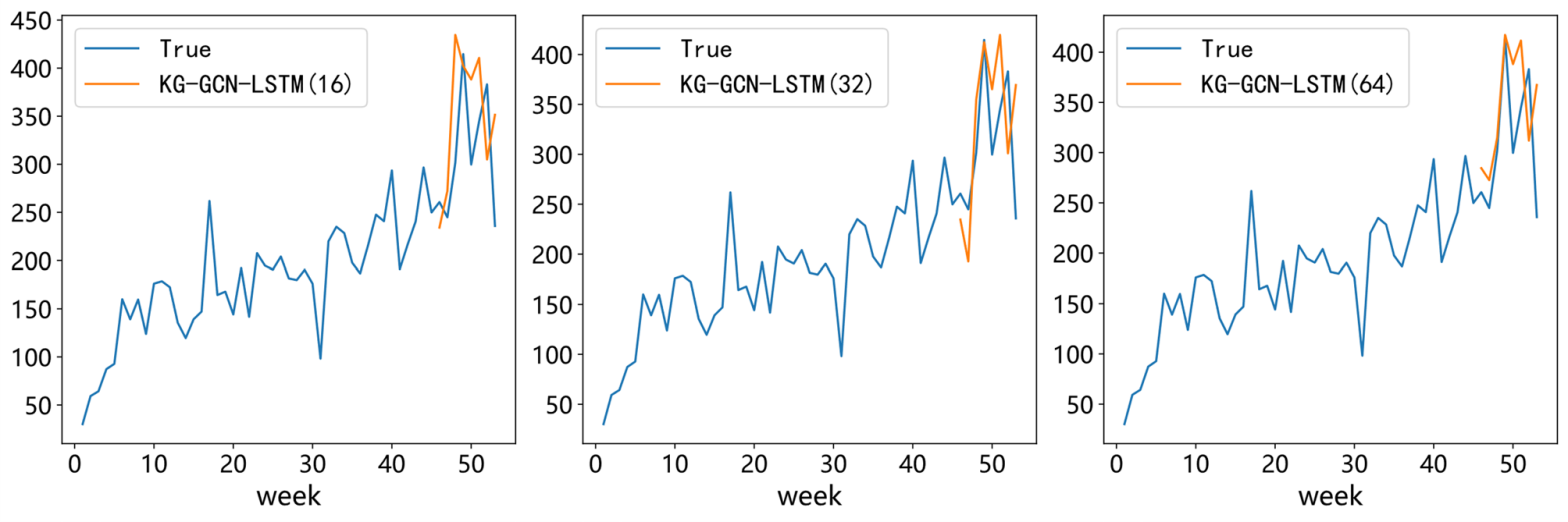
Comparison of true and predicted demand with different LSTM dimensions.

Figure 10 shows the trends of predicted values and actual values under different hidden sizes. In all three cases, the predicted trend is generally close to the actual trend, but the amount of deviation is different. When the hidden size is 16, the predicted curve reacts slower than the actual curve, so the deviation is larger. When the hidden size is 32, the prediction around week 46 is close to the actual value, but the deviation becomes larger in later weeks. When the hidden size is 64, the deviation after week 47 is smaller than that of size 32, which means increasing the hidden size can improve prediction accuracy.

In conclusion, the hidden size of LSTM has an obvious impact on the model performance. If the hidden size is too small, the ability of the model to express time series patterns is limited. Increasing the hidden size properly can help the model better describe the trend of pharmaceutical demand, and thus produce more accurate forecasts.

**Table 6:** Performance comparison of different LSTM hidden layer size.

Model	MAE	RMSE	SMAPE (%)
KG-GCN-LSTM(16)	68.4073	79.7613	10.36%
KG-GCN-LSTM(32)	61.1987	71.3250	9.91%
KG-GCN-LSTM(64)	53.9080	65.4058	8.24%

### Ablation study of components and data source

To comprehensively assess the contributions of various components and data sources in the proposed KG-GCN-LSTM model, we performed an ablation study by methodically eliminating or substituting essential modules. The objective of this investigation is to ascertain the individual and collective impacts of temporal modeling, graph-based relational learning, and knowledge graph enrichment on forecasting ability.

## Analysis at the component level

First, we looked at how the GCN and LSTM modules worked. In particular, swapping out the LSTM module for a one-layer MLP with 64 hidden units creates the KG-GCN-MLP version. On the other hand, taking off the GCN module leaves with a solo LSTM model.

The results of Table 7 demonstrate the synergistic importance of both components. The most dismal performance of the standalone LSTM variation is observed (MAE = 72.6728, RMSE = 84.4461, SMAPE = 11.21%), indicating that LSTM is inadequate for capturing the evolution of pharmaceutical demand over time. The KG-GCN-MLP achieves superior results (MAE = 65.2841, RMSE = 80.7454, SMAPE = 10.32%), demonstrating that the relation can detect graph patterns but still lacks temporal modeling.

The full KG-GCN-LSTM, on the other hand, does far better than both ablated versions on all measures (MAE = 53.9080, RMSE = 65.4058, SMAPE = 8.24%).

**Fig. 11:**
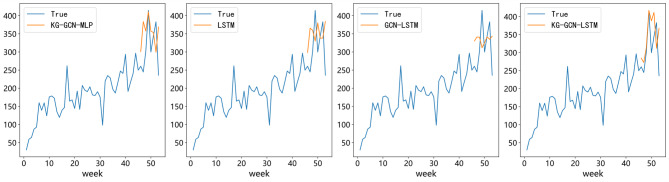
Comparison of true and predicted demand with ablation model.

Figure 11 shows that the LSTM model does a good job of capturing general demand trends, while the KG-GCN-MLP model shows how demand changes in the short term. The KG-GCN-LSTM model makes a more accurate and balanced prediction by combining both relational and temporal learning abilities.

These results show that the GCN and LSTM modules work together in different ways. The GCN effectively encodes relational knowledge from the pharmaceutical knowledge graph, capturing associations between drugs and symptoms. The LSTM captures long-term temporal dependencies and changes in drug use over time. By combining them, the KG-GCN-LSTM model can make the most accurate and reliable predictions in all experimental conditions.

## Analyzing the contribution of the knowledge graph

To further confirm the effect of the knowledge graph, a new baseline model called GCN-LSTM was tested. This model only uses the raw pharmaceutical demand time series as input. The graph only has one node that represents the target drug, and it doesn’t use any relational data from the knowledge graph like KG-GCN-LSTM does. This architecture lets us separate the effect of architectural design on performance from the effect of knowledge graph data.

As show in Table 6, the GCN-LSTM model has a SMAPE that is 1.36% lower than the LSTM model, and the KG-GCN-LSTM model has a SMAPE that is 2.97% lower than the LSTM model. These results show that adding graph-structured data from the knowledge graph accounts for about 1.61% of the performance gain, while the KG-GCN-LSTM model’s own architectural design accounts for the other 16% of the improvement.

In general, these results show that the better performance of KG-GCN-LSTM is due to the combined effects of data enrichment and structural innovation. The addition of the pharmaceutical knowledge graph helps the model grasp how medications and symptoms are related in terms of meaning, and the graph-based architecture makes it better at modeling complicated temporal-relational dynamics. These improvements all help make pharmaceutical demand forecasting more accurate, which is in line with the goal of developing strong healthcare supply chains.

**Table 7:** Ablation study on the contributions of GCN and LSTM in KG-GCN-LSTM.

Model	MAE	RMSE	SMAPE (%)
KG-GCN-MLP	65.2841	80.7454	10.32%
LSTM	72.6728	84.4461	11.21%
GCN-LSTM	61.6119	72.4715	9.85%
KG-GCN-LSTM	53.9080	65.4058	8.24%

### Discussion

In this study, we design a pharmaceutical demand forecasting model named KG-GCN-LSTM, which combines a knowledge graph with deep learning techniques. Experimental results show that the proposed model performs better than traditional statistical methods such as AutoARIMA, as well as commonly used machine learning and deep learning algorithms, including SVR, XGBoost, RNN, CNN-LSTM and NBEATS, while achieving performance comparable to TimeMixer. The KG-GCN-LSTM model introduces a pharmaceutical knowledge graph as an additional data source, where the GCN is used to extract the relational features between a drug and its related drugs, and the LSTM is employed to capture the nonlinear temporal patterns of demand. By integrating these two types of information, the model achieves more accurate demand forecasts.

To verify the effectiveness of the KG-GCN-LSTM design, we conducted a series of ablation experiments. First, we tested GCN models with different numbers of layers and examined the effect of the clip operation. The results show that using two layers and adding the clip operation leads to the best performance. The two-layer GCN can effectively extract association features among drugs, and applying clip to the second layer helps reduce the interference of related drugs on the target drug. Second, we tested LSTM models with different hidden sizes. The results indicate that the model performance improves as the hidden size increases, suggesting that a larger hidden size can better capture richer trend information in the demand series. Finally, we performed component-level and data-source-level ablation studies. The results show that removing either the GCN module or the LSTM module leads to worse performance, and removing the knowledge graph data also causes performance degradation. These results demonstrate that adding the knowledge graph provides more useful information for the model, and the GCN and LSTM modules work together: the GCN extracts relational features between drugs, while the LSTM extracts temporal features, and their combination enables more accurate demand prediction.

Compared with existing research on pharmaceutical demand forecasting, most current studies mainly rely on CNN, LSTM, Attention, Informer, and other single or hybrid models^45^. However, these methods only consider the information from the time series itself, and do not incorporate the relationships among drugs, such as substitution or combination relations. In real-world scenarios, the demand for one drug is often influenced by its substitute or related drugs. By integrating a knowledge graph into the forecasting model, KG-GCN-LSTM effectively improves prediction accuracy. Higher demand forecasting accuracy helps reduce stockout risk for pharmacies, and at the same time avoids excessive inventory, thus improving supply chain efficiency and reducing operational costs.

While our proposed model demonstrates superior performance, there are several limitations worth noting:

1. Dependency on Knowledge Graph Quality and Construction Cost: The performance of KG-GCN-LSTM heavily relies on the quality of the underlying Knowledge Graph. Incomplete or noisy relational data can degrade the model’s ability to capture semantic dependencies. Furthermore, aligning the Knowledge Graph with specific pharmaceutical items requires substantial manual verification and data cleaning. When applied to large-scale pharmaceutical datasets, this data preparation process becomes highly labor-intensive and time-consuming.
2. Limitation of Univariate Input: Currently, the model utilizes only historical time series values as input. It disregards external covariates—such as weather conditions, holidays, and epidemiological trends—which are known to significantly influence pharmaceutical demand. By ignoring these multi-source features, the model may fail to capture demand surges driven by external environmental changes.
3. Reliance on Manual Hyperparameter Tuning: Key hyperparameters of the model (e.g., learning rate, hidden layer dimensions) are currently determined based on empirical experience. This manual selection process cannot guarantee optimal model performance, and the trial-and-error approach for validating the best hyperparameters is computationally inefficient.

## Conclusions

This study focuses on improving the accuracy of pharmaceutical demand forecasting. Traditional statistical and machine learning methods often cannot capture both the complex temporal patterns of drug demand and the relationships among different drugs at the same time. Because of this limitation, forecasting errors may lead to problems such as inefficient inventory control or drug shortages.

To address these issues, we propose the KG-GCN-LSTM forecasting framework, which integrates relational features and temporal information to produce more accurate predictions. In this framework, a pharmaceutical knowledge graph is first introduced, and a clipped GCN is used to learn substitution relations and co-prescription patterns among drugs. These relational features are then combined with an LSTM network to model the nonlinear demand trends in historical sales data.

The experimental results show that KG-GCN-LSTM performs better than several baseline models, including AutoARIMA, SVR, XGBoost, RNN, CNN-LSTM, and NBEATS, across multiple evaluation metrics (MAE, RMSE, and SMAPE). Compared with the NBEATS model, our framework reduces SMAPE by 3.62%, demonstrating that it has higher prediction accuracy. Furthermore, while yielding performance comparable to TimeMixer, our model offers superior interpretability and is particularly well-suited for cold-start scenarios. Ablation studies also confirm the contribution of each module. About half of the performance improvement comes from the knowledge graph, which means that adding relational data can effectively enhance the prediction results.

Overall, the KG-GCN-LSTM framework not only achieves better performance than traditional and deep learning methods, but also provides a new way to apply knowledge graphs in pharmaceutical demand forecasting. The study shows that combining domain knowledge with neural network models can help address the complex challenges in medical supply chain prediction.

Although the proposed model performs well, there are still several directions for future work.

1. Integration of Multi-Source Heterogeneous Data: Currently, our model relies on univariate time series. Future research will incorporate external covariates—including weather, seasonality, holidays, epidemics, and demographic data—to capture complex demand drivers. To effectively process these high-dimensional inputs, we aim to adapt to state-of-the-art multivariate architectures such as TimeMixer or iTransformer^46^.
2. Automatic Hyperparameter Optimization: Instead of relying on empirical selection for hyperparameters (e.g., learning rate, hidden dimensions, dropout), future work will employ meta-heuristic algorithms, such as the Whale Optimization Algorithm^47^ (WOA). This will allow for adaptive and automated hyperparameter tuning, further maximizing the model’s predictive performance.

## Data Availability

The datasets generated and analyzed during the current study are available from the corresponding author on reasonable request.
